# Medical and surgical postoperative complications after breast conservation *versus* mastectomy in older women with breast cancer: Swedish population-based register study of 34 139 women

**DOI:** 10.1093/bjs/znac411

**Published:** 2022-12-13

**Authors:** Jana de Boniface, Robert Szulkin, Anna L V Johansson

**Affiliations:** Department of Surgery, Capio St. Göran’s Hospital, Stockholm, Sweden; Department of Molecular Medicine and Surgery, Karolinska Institutet, Stockholm, Sweden; SDS Life Science, Danderyd, Sweden; Department of Medical Epidemiology and Biostatistics, Karolinska Institutet, Stockholm, Sweden; Department of Medical Epidemiology and Biostatistics, Karolinska Institutet, Stockholm, Sweden; Cancer Registry of Norway, Oslo, Norway

## Abstract

**Background:**

Mastectomy rates in breast cancer are higher in older patients. The aim was to compare postoperative complication rates after breast-conserving surgery (BCS) to mastectomy in women aged 70–79 and older than 80 years *versus* those aged 50–69 years, and to evaluate survival effects.

**Methods:**

This population-based cohort included women aged 50 years and older with invasive breast cancer T1–3 N0–3 M0 operated on in Sweden 2008–2017. Major surgical and medical 30-day postoperative complications were assessed in adjusted logistic regression models. Overall survival was assessed in Cox models adjusted for clinical confounders, socio-economics, and comorbidity.

**Results:**

Of 34 139 women, 8372 (24.5 per cent) were aged 70–79 years, 3928 (11.5 per cent) were 80 years of age or older, and 21 839 (64.0 per cent) were aged 50–69 years. Major surgical postoperative complications did not differ between age groups receiving equivalent surgery (BCS: 2.1 per cent and 2.0 per cent *versus* 2.1 per cent (*P* = 0.90); mastectomy: 4.6 per cent and 5.1 per cent *versus* 4.6 per cent (*P* = 0.49)). Major medical postoperative complications were higher in women aged >70 years than in women aged 50–69 years (BCS: 1.0 per cent and 2.3 per cent *versus* 0.4 per cent (*P* < 0.001); mastectomy: 3.1 per cent and 6.2 per cent *versus* 1.1 per cent (*P* < 0.001)), which persisted after adjustments. In women treated by mastectomy, major medical and surgical postoperative complications were associated with worse overall survival in all but the middle age group.

**Conclusion:**

Mastectomy has higher medical and surgical postoperative complication rates than BCS. Major medical postoperative complications increase significantly with age. Major postoperative complications are associated with worse survival after mastectomy, which should be used with caution in older women.

## Teaser

Older women (aged 70 years or older) with breast cancer are not more prone to major surgical postoperative complications than women aged 50–69 years, but they experience more major medical postoperative complications. Older women are more commonly operated on with mastectomy, which comes with an increased risk of complications, associated with survival. Mastectomy should be considered with caution in older patients with breast cancer.

## Introduction

About a third of patients with newly diagnosed breast cancer are 70 years of age or older. In Sweden, this proportion increased from 29 per cent in 2010 to 34 per cent in 2020^1^. Age is a major risk factor for developing breast cancer, and, with an ageing population, a further increase in the number of older women with breast cancer should be expected.

Breast cancer treatment in older patients deviates from that offered to others. This is partly due to accumulating comorbidities with age, aggravating the competing mortality risk present in older individuals. Therefore, treatment strategies in older adults need to balance the risk of a breast cancer recurrence against the risk of death of other causes, this being more strongly affected by frailty than chronological age itself. Breast cancer surgery is more often omitted in older women and replaced by primary endocrine treatment^2^. This strategy has however shown worse survival than up-front surgery, and is therefore only recommended in those with a life expectancy of less than 5 years^3–5^. With increasing age, the likelihood of receiving a mastectomy increases, while the likelihood of both being offered and of accepting breast reconstruction is significantly reduced^2,6^. The lower reconstruction rates are thought to be due to a combination of reduced patient demand and surgeons’ misconception of a higher risk of postoperative complications (POCs) with age, while the increasing mastectomy rates may be intended to decrease the need for adjuvant radiotherapy (RT) and the risk of a second surgery for positive margins^6^. Older women are more likely to be offered mastectomy without adjuvant RT instead of breast-conserving surgery (BCS) with whole-breast irradiation compared to women aged 51–65 years^7^. However, it is unclear how appropriate this strategy is when considering the combination of a potentially elevated risk of POCs and the increasing prevalence of frailty in the older population.

Postoperative morbidity and mortality rates are higher in older adults after major surgery such as cholecystectomy and oesophagectomy^8,9^. However, after breast surgery, including major breast reconstruction, complication rates in older individuals are not increased^10,11^. Importantly, even more extensive oncoplastic breast-conserving procedures such as therapeutic mammaplasty have lower major complication rates (2.1 per cent) than mastectomy without (5.0 per cent) or with breast reconstruction (14.4 per cent)^12^. A negative impact of POCs on survival rates has been shown for other types of cancer such as colorectal, gastric, lung, and head and neck cancer^13^. In breast cancer, the results have been equivocal. A significant association was recently confirmed in a publication from the Swedish National Quality Register for Breast Cancer^14,15^.

The present population-based cohort study aimed to assess the incidence of POCs in older patients following the most common breast surgical interventions in this age group, namely BCS with adjuvant RT and mastectomy without RT, and evaluate potential associations with survival.

## Methods

This cohort study included prospectively collected nationwide data from the Swedish National Quality Register for Breast Cancer (NKBC), which has harmonized online reporting since 2008 and is considered to be 98–99 per cent complete. A recent validation study concluded that register data are in exact agreement with source data in more than 90 per cent of variables selected for validation^16^. The NKBC includes detailed clinical information on patient factors, tumour characteristics, and treatments.

Data on women with invasive breast cancer who underwent breast surgery with a known surgery date between 1 January 2008 and 31 December 2017, known tumour size (T1–3), any nodal status (N0–3), and available data on planned or given adjuvant radiotherapy (RT) were obtained from the NKBC. Using the personal identification number assigned to all Swedish residents, the study cohort was individually linked to several Swedish health and population registers. All diagnoses from inpatient and outpatient care were received from the National Patient Register of the Swedish National Board of Health and Welfare; information on date and cause of death from the Cause of Death Register; and data on socio-economic factors, education, and country of birth from demographic registers at Statistics Sweden. Subsequently, women with an unclear date of surgery or reused personal identification number, and also one woman with a recorded death before 2008, were excluded. For the present analysis, only women aged 50 years or older who had been treated by BCS with RT, or mastectomy without immediate breast reconstruction and without RT, and without any cancer-related reoperation were retained. Women not receiving whole-breast irradiation after BCS were also not included since this treatment was not in accordance with Swedish treatment guidelines at the time and often chosen due to significant comorbidity. The aim was to compare age groups within treatment categories that are typical treatment alternatives for older adults with early-stage breast cancer, namely mastectomy without adjuvant RT and BCS with RT. Seventeen women who died within 30 days of surgery were excluded as they were not at risk for POCs for a full 30 days (*Fig. S1*).

### Major surgical and medical postoperative complications

Major surgical POCs were defined as having at least one registered diagnosis or procedure code of bleeding or wound complication (ICD-10 codes: T810, T811, T817, HWD00, HWE00, HWA00, T813, HWF00), infection (T857, T814, HWB00, HWC00), and/or unspecified complications (T854, T856, T858, T859, T812, T815, T818, T818W, T819, HWW99, T889) in connection with the index inpatient episode or as a new inpatient episode (readmission) within 30 days of their first surgery. Major medical POCs were determined using the ICD-10 codes for severe cardiac (I630-9, I210-9, I26, I500-9) and respiratory events (J150-9, J180-9, J69, J80, J93, S27; *Table S1*).

### Tumour characteristics

Tumour size (T) and nodal status (N) were defined based on information from histopathology of the surgical specimen, or on clinical and/or radiological examination in the case of neoadjuvant systemic chemotherapy, using the eighth edition of the AJCC Cancer Staging Manual. Prognostic groups were defined as T1N0, T1N1, T1N2, T2N0, T2N1, T2N2, T3N0–2 and T1–3N3. Tumour biology, that is expression of oestrogen receptor (ER), progesterone receptor (PR), and human epidermal growth factor receptor 2 (HER2), including confirmative *in situ* hybridization tests in case of 2 + on immunohistochemistry, was based on pretreatment core needle biopsy if neoadjuvant chemotherapy was given, and on the surgical specimen otherwise. ER and PR were defined as negative if less than 10 per cent of cells were stained for the respective receptor on immunohistochemistry, and cases were hormone receptor-positive (HR+) if ER + and/or PR + and hormone receptor-negative (HR−) if ER− and PR−. Subtypes were HR + HER2−, HR + HER2+, HR−HER2+, and HR−HER2−.

### Treatment

Primary treatment was either primary surgery or neoadjuvant systemic treatment. Locoregional treatment was categorized as BCS + RT or mastectomy without RT (mastectomy – RT). The use of oncoplastic techniques was not specifically considered. Since the exact target and dose of RT were unavailable, postoperative RT was entered as a binary option (yes/no). In the relevant time frame, regional RT was indicated in case of lymph node metastases, regardless of the type of axillary surgery performed, except in cases of micrometastases only or one macrometastasis in grade 1 tumours after BCS. A positive clinical nodal status before neoadjuvant systemic treatment always required postoperative regional RT. Postmastectomy RT was endorsed in T1N0 and T2N0 tumours in case of extensive multifocality; however, in the register, only the size of the largest tumour focus is reported, in accordance with TNM classification.

Final axillary surgery was defined as sentinel lymph node biopsy or axillary lymph node dissection. Registered neoadjuvant and adjuvant systemic treatment included chemotherapy, endocrine treatment, and anti-HER2 therapy.

### Comorbidities and socio-economic background

Comorbidities were identified according to the Royal College of Surgeons’ Charlson Comorbidity Index^17^, and included main and contributing diagnoses of any listed comorbidity in the National Patient Register in the 12 months before the first breast cancer treatment. The highest educational level (categorized as 9 years or less (primary), 10–13 years (secondary), and 13 years and older (tertiary)) and family income (categorized as low (Q1: 0 per cent to 25 per cent), middle (Q2-Q3: more than 25 per cent to 75 per cent) and high (Q4: more than 75 per cent to 100 per cent)) up to 1 year prior to a breast cancer diagnosis was recorded. Income was adjusted for inflation during the study period. Country of birth was defined as Sweden, Europe except Sweden, and countries outside of Europe. Data were available from 2008 to 2017, meaning that for patients diagnosed with breast cancer in 2008, information on comorbidities and socio-economic background from 2008 was used.

### Ethics

The study was approved by the Ethical Review Authority in Sweden (2017/2493–31), which allows for the use of register data without individual informed consent for research purposes.

### Statistical methods

The distributions of patient, tumour, and treatment variables between age groups (50–69 years, 70–79 years, and 80 years and older) were compared with the χ^2^ test for categorical variables, and an ANOVA F-test for continuous variables.

The occurrence of major POCs within 30 days was treated as a binary variable. The association between age group and POCs within levels of locoregional treatment (BCS + RT and mastectomy – RT, respectively) was estimated as odds ratios (OR) with 95 per cent confidence intervals using logistic regression. The models were adjusted for year and region of diagnosis, primary treatment, education, family income, country of birth, and Charlson Comorbidity Index.

The impact of major POCs on survival was assessed within levels of age and locoregional treatment. For the survival analysis, the follow-up started at the date of surgery and ended at the date of death or the end of the study in September 2019, whichever came first. Overall survival was defined as death due to any cause, while breast cancer-specific survival included death due to breast cancer while censoring for other causes of deaths. Five- and 10-year survival proportions were estimated with the Kaplan–Meier method and compared with log-rank tests. Stratified Cox proportional hazards regression estimated hazard ratios (HR) with 95 per cent confidence intervals for the association between overall survival and POCs, with models including stratification for prognostic group (TN stage), histological grade, tumour subtype, endocrine therapy, and locoregional treatment, and covariate adjustments for year and region of diagnosis, chemotherapy, education, family income, country of birth, and Charlson Comorbidity Index. All Cox models included an interaction between age, locoregional treatment, and POCs. Survival analyses are presented for major surgical and major medical POCs separately. Owing to few events in the relevant subgroups, Cox regression for breast cancer-specific survival was not estimated.

The significance level was 5 per cent and all tests were two-sided. In the adjusted analyses, women with missing information on any variable were excluded. All statistical analyses were performed using R version 4.1.2 (R Foundation for Statistical Computing, Vienna, Austria).

## Results

Median duration of follow-up was 6.14 (range 0.09–11.70) years. Seventeen women had died within 30 days of surgery, nine in the 80 years or older group, six in the 70–79 years group, and two in the 50–69 years age group, with registered causes of death being ischaemic heart disease in three, suicide in one, Parkinson’s disease in one, breast cancer in nine, cerebral ischaemia in one, chronic obstructive pulmonary disease in one, and metabolic disorder in one, and were not included in this analysis. Fifteen of these 17 women had been treated by mastectomy. Of the remaining 34 139 women, 8372 (24.5 per cent) were aged 70–79 years, 3928 (11.5 per cent) 80 years or older, and 21 839 (64.0 per cent) were aged 50–69 years. Mastectomy without adjuvant radiotherapy (mastectomy – RT) was recorded in 2968 in women aged 70–79 years (35.5 per cent) and in 3279 women aged 80 years or older (83.5 per cent), and in 4077 women in the 50–69 year age group (18.7 per cent; *P* < 0.001). Larger tumours, node positivity, and thus axillary lymph node dissection were most prevalent in women aged 80 years or older (*P* < 0.001, *Table S2*). The proportion of lobular carcinomas was higher in the older age groups (*P* < 0.001). Luminal tumour subtypes were least common in the oldest age group (*P* < 0.001), with no corresponding decrease in endocrine treatment (*P* < 0.001) but less chemotherapy use (*P* < 0.001). In terms of socio-economic background, women in the older age groups were more often born in Sweden, living in a single household with a low family income, and more often had nine years or less of education (all *P* < 0.001). The proportion with no comorbidities was largest among the women aged 50–69 years (*P* < 0.001).

The occurrence of major surgical POCs did not differ between the age groups when receiving the same type of breast surgery (BCS, *P* = 0.900; mastectomy, *P* = 0.490; *Table 1*) but was significantly more frequent after mastectomy than after BCS in all age groups (*P* < 0.001). As presented in *Table 1*, major medical POCs increased with age after BCS and after mastectomy (*P* < 0.001), and were more common after mastectomy than after BCS in all age groups (all *P* < 0.001). After adjustment for year of surgery, region of residence, primary treatment modality, education level, country of birth, family income, and Charlson Comorbidity Index, age group was not statistically significantly associated with major surgical POCs after BCS (OR 70–79 years: 0.93 (95 per cent c.i. 0.74 to 1.16); OR 80 years and older: 0.91 (95 per cent c.i. 0.49 to 1.53)) or after mastectomy (OR 70–79 years: 0.94 (95 per cent c.i. 0.74 to 1.19); OR 80 years or older: 1.05 (95 per cent c.i. 0.83 to 1.32)). However, the older groups had a significantly higher risk than the reference group (50–69 years) of suffering a major medical POC after BCS (OR 70–79 years: 2.02 (95 per cent c.i. 1.40 to 2.89); OR 80 years or older: 3.90 (95 per cent c.i. 2.12 to 6.73)) and after mastectomy (OR 70–79 years: 2.10 (95 per cent c.i. 1.44 to 3.09; OR 80 years or older: 3.74 (95 per cent c.i. 2.65 to 5.39)).

**Table 1:** Odds ratios (ORs) of major surgical and major medical postoperative complications (POCs) within 30 days according to locoregional treatment (BCS + RT *versus* mastectomy – RT) and age group

	No major surgical POC	Major surgical POC	With vs without major surgical POC, OR (95% c.i.)*	No major medical POC	Major medical POC	With *versus* without major medical POC, OR (95% c.i.)*
**BCS + RT**						
ȃ50–69 years	17 381 (97.9)	381 (2.1)	1.00 (ref)	17 686 (99.6)	76 (0.4)	1.00 (ref.)
ȃ70–79 years	5293 (97.9)	111 (2.1)	0.93 (0.74–1.16)	5349 (99.0)	55 (1.0)	2.02 (1.40–2.89)
ȃ80+ years	636 (98.0)	13 (2.0)	0.91 (0.49–1.53)	634 (97.7)	15 (2.3)	3.90 (2.12–6.73)
**Mastectomy – RT**						
ȃ50–69 years	3890 (95.4)	187 (4.6)	1.00 (ref.)	4032 (98.9)	45 (1.1)	1.00 (ref.)
ȃ70–79 years	2832 (95.4)	136 (4.6)	0.94 (0.74–1.19)	2877 (96.9)	91 (3.1)	2.10 (1.44–3.09)
ȃ80+ years	3111 (94.9)	168 (5.1)	1.05 (0.83–1.32)	3077 (93.8)	202 (6.2)	3.74 (2.65–5.39)

Values are *n* (%) unless otherwise stated. *Adjusted for year, region of diagnosis, primary treatment, education, family income, country of birth, and comorbidity index. BCS, breast-conserving surgery; RT, radiotherapy.

**Fig. 1:**
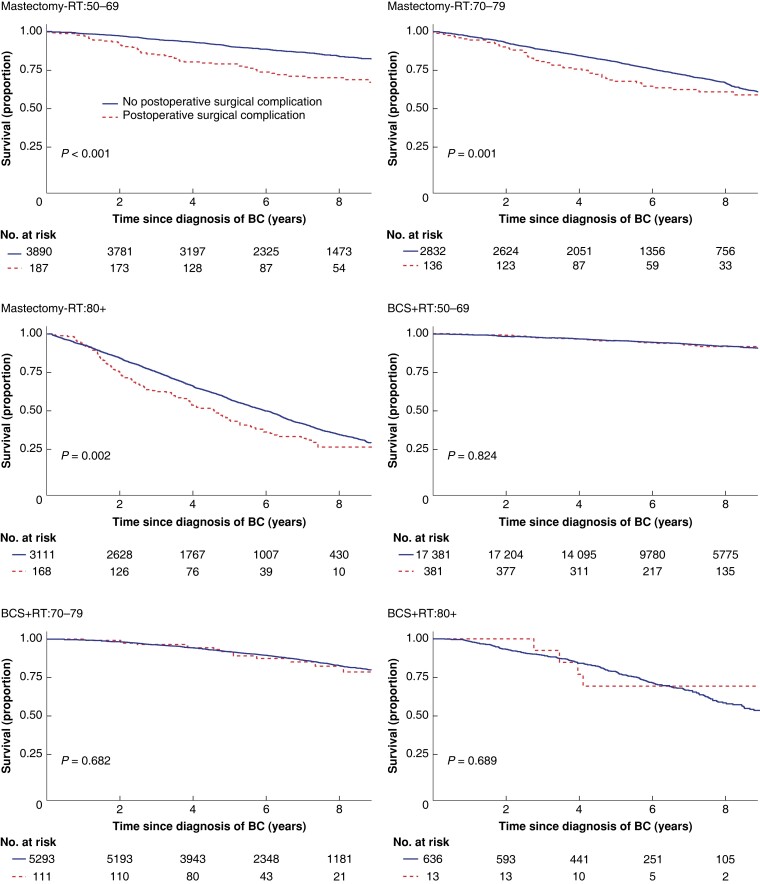
Unadjusted overall survival by locoregional treatment and age in women with breast cancer (BC) with and without at least one major surgical postoperative complication within 30 days Upper left: mastectomy (Mx) without radiotherapy (RT) (Mx-RT), 50–69-year-olds; upper right: Mx-RT, 70–79-year-olds; middle left: Mx-RT, patients aged 80 years or older; middle right: breast-conserving surgery (BCS) + RT, 50–69-year-olds; lower left: BCS + RT, 70–79-year-olds; lower right: BCS + RT, patients aged 80 years or older.

**Fig. 2:**
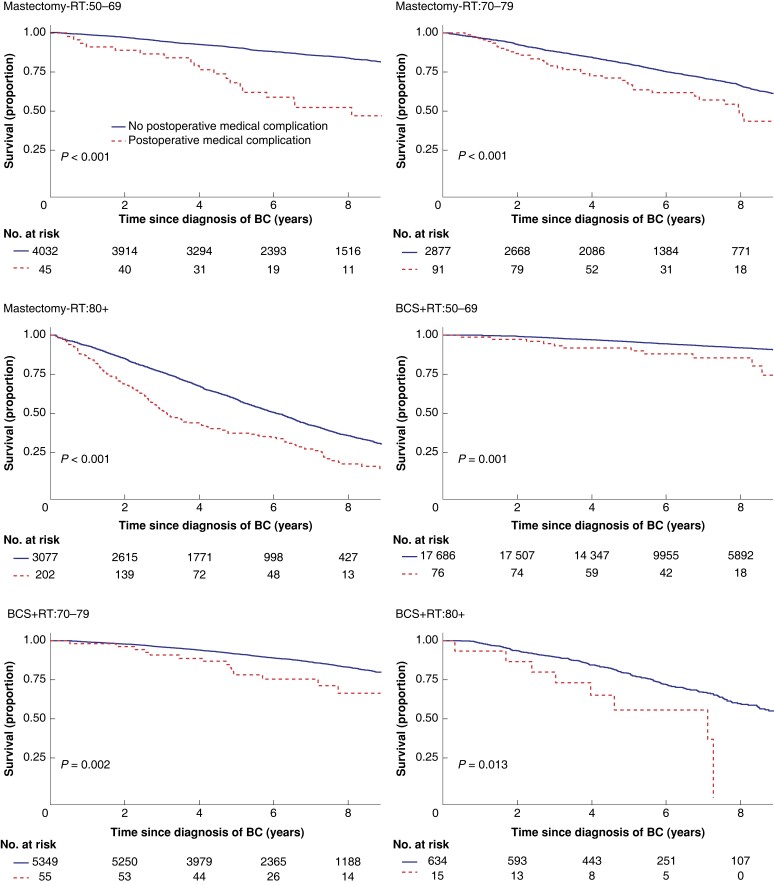
Unadjusted overall survival by locoregional treatment and age in women with breast cancer (BC) with and without at least one major medical postoperative complication within 30 days Upper left: mastectomy (Mx) without radiotherapy (RT) (Mx-RT), 50–69-year-olds; upper right: Mx-RT, 70–79-year-olds; middle left: Mx-RT, patients aged 80 years or older; middle right: breast-conserving surgery (BCS) + RT, 50–69-year-olds; lower left: BCS + RT, 70–79-year-olds; lower right: BCS + RT, patients aged 80 years or older.

Women suffering a major surgical POC had lower crude overall survival in all age groups after mastectomy but not after BCS (*Table 2*, *Fig. 1*). In the adjusted analyses, having a major surgical POC was significantly associated with worse overall survival only after mastectomy in the oldest and youngest age groups (HR 50–69 years: 1.92 (95 per cent c.i. 1.35 to 2.74; HR 80 years or older: 1.43 (95 per cent c.i. 1.09 to 1.86), *Table 2*). Women experiencing a major medical POC had lower overall survival in all age groups after both types of breast surgery (*Table 3*, *Fig. 2*). After adjustments, major medical POCs were statistically significantly associated with worse overall survival only in the oldest age group after mastectomy (HR 1.60, 95 per cent c.i. 1.25 to 2.06; *Table 3*).

**Table 2:** All-cause death by locoregional treatment (BCS + RT *versus* mastectomy – RT), age group, and at least one major surgical postoperative complication (POC) within 30 days

	Overall survival			
	5-year survival, % (95% c.i.)	10-year survival, % (95% c.i.)	HR (95% c.i.)*	*P* value
**BCS + RT**				
ȃ50–69 years				
ȃȃNo major surgical POC	96 (96–96)	89 (89–90)	1.00 (ref.)	
ȃȃMajor surgical POC	95 (93–97)	88 (83–94)	1.12 (0.74–1.68)	0.594
ȃ70–79 years				
ȃȃNo major surgical POC	92 (91–93)	76 (74–78)	1.00 (ref.)	
ȃȃMajor surgical POC	92 (86–97)	72 (58–89)	0.92 (0.49–1.73)	0.796
ȃ80 + years				
ȃȃNo major surgical POC	79 (75–82)	45 (39–53)	1.00 (ref.)	
ȃȃMajor surgical POC	69 (48–99)	69 (48–99)	1.35 (0.46–3.95)	0.580
**Mastectomy – RT**				
ȃ50–69 years				
ȃȃNo major surgical POC	91 (90–92)	80 (78–82)	1.00 (ref.)	
ȃȃMajor surgical POC	79 (73–85)	65 (57–74)	1.92 (1.35–2.74)	<0.001
ȃ70–79 years				
ȃȃNo major surgical POC	80 (79–82)	56 (53–59)	1.00 (ref)	
ȃȃMajor surgical POC	68 (60–77)	45 (33–60)	1.21 (0.82–1.78)	0.327
ȃ80 + years				
ȃȃNo major surgical POC	58 (56–60)	23 (21–26)	1.00 (ref.)	
ȃȃMajor surgical POC	45 (37–53)	26 (19–36)	1.43 (1.09–1.86)	0.009

Adjusted for year, region of diagnosis, prognostic group, grade, subtype, chemotherapy, endocrine therapy, education, family income, country of birth, and comorbidity index. BCS, breast-conserving surgery; RT, radiotherapy; HR, hazard ratio.

**Table 3:** All-cause death by locoregional treatment (BCS + RT *versus* mastectomy – RT), age group, and at least one major medical postoperative complication (POC) within 30 days

	Overall survival			
	5-year survival, % (95% c.i.)	10-year survival, % (95% c.i.)	HR (95% c.i.)*	*P* value
**BCS + RT**				
ȃ50–69 years				
ȃȃNo major medical POC	96 (96–96)	89 (89–90)	1.00 (ref.)	
ȃȃMajor medical POC	92 (86–98)	75 (60–93)	1.59 (0.80–3.16)	0.185
ȃ70–79 years				
ȃȃNo major medical POC	92 (91–93)	76 (74–79)	1.00 (ref.)	
ȃȃMajor medical POC	78 (68–91)	59 (43–83)	1.72 (0.92–3.20)	0.090
ȃ80 + years				
ȃȃNo major medical POC	79 (76–83)	47 (40–54)	1.00 (ref.)	
ȃȃMajor medical POC	56 (34–91)	0 (NA)	1.08 (0.31–3.74)	0.908
**Mastectomy – RT**				
ȃ50–69 years				
ȃȃNo major medical POC	91 (90–91)	80 (78–81)	1.00 (ref.)	
ȃȃMajor medical POC	68 (55–84)	47 (32–69)	1.91 (0.99–3.67)	0.052
ȃ70–79 years				
ȃȃNo major medical POC	80 (79–82)	56 (53–59)	1.00 (ref.)	
ȃȃMajor medical POC	67 (57–78)	24 (11–52)	1.25 (0.81–1.93)	0.308
ȃ80 + years				
ȃȃNo major medical POC	58 (57–60)	24 (21–26)	1.00 (ref.)	
ȃȃMajor medical POC	37 (31–45)	13 (8–22)	1.60 (1.25–2.06)	<0.001

Adjusted for year, region of diagnosis, prognostic group, grade, subtype, chemotherapy, endocrine therapy, education, family income, country of birth and comorbidity index. BCS, breast-conserving surgery; RT, radiotherapy; HR, hazard ratio; NA, not available.

When specifically assessing 24 432 women with T1 tumours, in order to evaluate major POC rates and survival effects in patients who would have been potential candidates for BCS, the increased risk of major medical POCs was confirmed for both older groups after BCS (OR 70–79 years: 1.62 (95 per cent c.i. 1.04 to 2.48); OR 80 years or older: 3.57 (95 per cent c.i. 1.61 to 7.05) and after mastectomy (OR 70–79 years: 2.21 (95 per cent c.i. 1.35 to 3.71); OR 80 years or older: 3.33 (95 per cent c.i. 2.05 to 5.56)). The association of major medical POCs with overall survival was confirmed in women aged 80 years or older with T1 tumours after mastectomy (HR 1.63, 95 per cent c.i. 1.07 to 2.47).

## Discussion

In this analysis of a large cohort from a prospectively maintained population-based register, major medical but not major surgical POCs were more common in women aged 70 years or older than in those aged 50–69 years. Mastectomy significantly increased the risk of both major surgical and major medical POCs. Major medical and surgical POCs were associated with worse overall survival after mastectomy in women aged 80 years and older and those aged 50–69 years.

One frequent argument for the choice of mastectomy without RT over BCS and postoperative RT is the omission of RT. RT implies a significant number of daily visits to the hospital which may be problematic, especially for older adults in rural areas, where travel to healthcare institutions can be a substantial obstacle^18^. In addition, older women and their surgeons may be more prone to consider the loss of a breast as less traumatic than for younger patients^19^. It is however reported that the benefits of BCS regarding quality of life and body image are also significant in older women, and several observational studies have reported a better overall and breast cancer-specific survival after BCS than after mastectomy^20–22^. Adding the fact that the routine time frame for RT after BCS has recently been shortened from about 5 weeks to 1 week as a result of the FAST-Forward trial, the omission of RT should no longer be an argument for denying older women BCS^23^.

In an international comparison, the major POC rate was low in the present cohort. According to a prospective cohort study published by the GlobalSurg Collaborative, 30-day major surgical complications (Clavien–Dindo grade III–V) after breast cancer surgery occurred in 5.9 per cent of patients^24^. POCs may directly impact postoperative mortality; in the present cohort, the 30-day mortality rate of 0.05 per cent was low in comparison with the above-mentioned global analysis, which reported 0.2 per cent. Nevertheless, mortality rates may increase due to major medical POCs such as cardiac or respiratory events in aggravated or newly acquired conditions. Furthermore, several studies have pointed toward a role of POCs in cancer-specific survival, which was recently confirmed in breast cancer^15^. In the present analysis, unadjusted overall survival proportions were negatively affected by major medical POCs and by major surgical POCs after mastectomy, but after adjustment for a wide range of clinical and socio-economic factors, an association of major POCs with survival persisted only in subgroups. This may support the notion that contributing factors such as comorbidity and socio-economic status are associated with the selection of the type of surgery performed and survival.

In older women with tumours small enough to be appropriately treated by BCS and postoperative RT, there are few arguments left to recommend mastectomy without RT. Considering the significantly increased risk of both major surgical and major medical POCs, and adding the well-documented benefit of less extensive and less traumatic surgery from a patient perspective, as well as the growing body of evidence for a survival benefit of BCS + RT over mastectomy – RT, the first recommendation of breast cancer surgery for older and often more frail individuals should be breast conservation.

In a previous report from the same Swedish national register, mastectomy with or without immediate breast reconstruction was more common than BCS in women older than 65 years of age and in women of lower socio-economic status^7^. Since breast reconstruction is less commonly performed in older and socio-economically weaker women, the present report excluded cases of immediate breast reconstruction^25,26^. In addition, the present analysis did not include women treated by mastectomy and postoperative RT, since it is a common clinical observation that older women receive mastectomy instead of BCS, even for small node-negative tumours. The increased proportion of mastectomy without RT in the oldest age group has already been reported in another publication from the same population-based cohort^22^.

The main strengths of the present report are the high data quality and external validity in a national population-based register. A number of potential confounders for both POCs and survival outcomes, such as smoking and BMI, were not however available from national registers. It may be suspected that such factors are partly embedded in socio-economic status. Additionally, any delay of adjuvant treatment initiation could not be assessed. Such a delay could have been more common in cases of major POCs, thus contributing to worse survival outcomes. Also, disease recurrence is not fully covered in the available national registers and could not be assessed in this analysis. Cause-specific survival in older individuals may be prone to bias due to unreliable cause-of-death information, while overall survival will not be affected by such misclassification. However, such bias is lower in patients younger than 80 years^27^.

It is likely that women undergoing mastectomy as a second or third surgery, that is a completion mastectomy after BCS rendering positive margins, have a higher risk of POCs than those undergoing mastectomy as a first procedure. Here, women with more than one cancer-related breast surgery were excluded in order to provide a cleaner comparison of two surgical methods. It should thus be considered that the presented POC rates may neither be representative of BCS consisting of repeated surgeries nor for completion mastectomy after attempted BCS.

Rather than chronological age, frailty should be used for the assessment of treatment-related risks in older adults^4^. Frailty comprises factors such as unintentional weight loss in the previous year, low grip strength, self-reported exhaustion, slow walking speed and low physical activity, and becomes more prevalent with age. Unfortunately, no data on frailty were available in the present cohort since the necessary factors are not recorded in any national registers. Instead, the relatively crude Charlson Comorbidity Index was used, allowing an estimation of existing diagnoses before breast cancer surgery but not taking deterioration in health status prior to, during, or after cancer treatment into account. Furthermore, the Charlson Comorbidity Index does not integrate all potentially relevant comorbidities, which may result in residual confounding. In order to mitigate this risk, data constituting the Charlson Comorbidity Index were extracted from both in- and outpatient health care, and both main and contributing diagnostic codes were considered.

Retrospective data offer a lower grade of evidence than that from the large randomized clinical trials performed in the 1970s and 1980s. Modern randomized trials on the topic of breast conservation *versus* mastectomy are however unlikely and ethically questionable. Therefore, prospective standardized data collection, including factors such as frailty, comorbidity, socio-economic background, and patient-reported outcomes should be strongly supported.

## Supplementary Material

znac411_Supplementary_Data

## Data Availability

The authors are willing to make their data, analytic methods, and study materials available to other researchers upon reasonable request, including relevant ethical and legal permissions to the corresponding author. The presented analysis was not pre-registered.
