# Supplementary material for: Medical and surgical postoperative complications after breast conservation versus mastectomy in older women with breast cancer: Swedish population-based register study of 34 139 women
Source: Br J Surg. 2022 Dec 13;110(3):344–52. doi: 10.1093/bjs/znac411 (PMC10364521; doi:10.1093/bjs/znac411)
Supplement: znac411_Supplementary_Data [file znac411_supplementary_data.docx]

**Medical and surgical postoperative complications after breast conservation versus mastectomy in older women with breast cancer: Swedish population-based register study in 34139 women**

Jana de Boniface Ph.D.^1,2^, Robert Szulkin Ph.D.^3,4^, Anna L.V. Johansson Ph.D.^4,5^

^1^ Department of Surgery, Capio St. Göran’s Hospital, Stockholm, Sweden

^2^ Department of Molecular Medicine and Surgery, Karolinska Institutet, Stockholm, Sweden

^3^ SDS Life Science, Danderyd, Sweden

^4^ Department of Medical Epidemiology and Biostatistics, Karolinska Institutet, Stockholm, Sweden

^5^ Cancer Registry of Norway, Oslo, Norway

*Author responsible for correspondence and to whom requests for reprints may be addressed*:

Jana de Boniface, MD, PhD

ORCID 0000-0001-9518-0902

Department of Surgery, Breast Center, Capio St. Göran’s Hospital, 11219 Stockholm, Sweden

[jana.de-boniface@ki.se](mailto:jana.de-boniface@ki.se)

Phone: +46 8 58701000

**Supplementary Materials - Index**

| **Supplementary Figures and Tables** |  |
| --- | --- |
| Figure S1 | *Page 1* |
| Table S1 | *Page 3* |
| Table S2 | *Page 4* |
|  |  |

**Figure S1**. CONSORT flow chart for study inclusion.

**Data extraction from NKBC 2008-2017**

**N=66490 invasive breast cancers**

**Exclusions before register linkages:**

- No breast surgery performed, N=134
- Type of breast surgery not reported, N=4917
- Distant metastasis at diagnosis and/or no surgery planned, N=162
- Locally advanced tumors (T4), N=572
- Clinical (in neoadjuvant cases) or pathological (in primary surgery cases) tumor stage not reported, N=1141
- Non-invasive breast cancer reported incorrectly to the register, N=1
- No information on planned or received adjuvant radiotherapy, N=854

**Exclusions after register linkages:**

- Deviating date of surgery, N=8
- Reused ID number, N=52
- Death date before 2008, N=1
- Age under 50 years, N=10397
- Immediate breast reconstruction or no information on reconstruction, N=1064
- More than one breast surgery performed, N=3305
- Breast-conserving surgery without radiotherapy or mastectomy with radiotherapy as locoregional treatment, N=8254
- Death within 30 days of surgery, N=17

**Included in analysis**

**N=34139**

**N=58709 breast cancers in 57237 women**

**Linkage to Patient Register, Cause of Death Register, and LISA database for socioeconomic background**

NKBC: Swedish National Breast Cancer Register

RT: radiotherapy

LISA: Longitudinal Integrated Database for Health Insurance and Labour Market Studies

**Table S1**. Disease codes from the International Statistical Classification of Diseases and Related Health Problems, Tenth Revision (ICD-10) and surgical intervention codes used to define major surgical and medical postoperative complications.

| **ICD-10 code** | **Definition** |
| --- | --- |
| *Major surgical postoperative complications* | |
| T810 | Haemorrhage and haematoma complicating a procedure, not elsewhere classified |
| T811 | Shock during or resulting from a procedure, not elsewhere classified |
| T812 | Accidental puncture and laceration during a procedure, not elsewhere classified |
| T813 | Disruption of operation wound, not elsewhere classified |
| T814 | Infection following a procedure, not elsewhere classified |
| T815 | Foreign body accidentally left in body cavity or operation wound following a procedure |
| T817 | Vascular complications following a procedure, not elsewhere classified |
| T818 | Disruption of operation wound, not elsewhere classified |
| T819 | Unspecified complication of procedure |
| T889 | Complication of surgical and medical care, unspecified |
| HWA00 | Reoperation for wound dehiscence after breast surgery |
| HWB00 | Reoperation for superficial infection after breast surgery |
| HWC00 | Reoperation for deep infection after breast surgery |
| HWD00 | Reoperation for superficial haemorrhage after breast surgery |
| HWE00 | Reoperation for deep haemorrhage after breast surgery |
| HWF00 | Reoperation for insufficient suture after breast surgery |
| HWW99 | Other reoperation on breast gland, unspecified |
| *Major medical postoperative complications* | |
| I630-9 | Cerebral infarction |
| I210-9 | Myocardial infarction |
| I26 | Pulmonary embolism |
| I500-9 | Cardiac failure |
| J150-9, J180-9 | Infectious pneumonia |
| J69 | Aspirational pneumonia |
| J80 | Respiratory failure and acute respiratory distress syndrome |
| J93 | Pneumothorax |
| S27 | Traumatic pneumothorax |

**Table S2.** Patient, treatment and disease characteristics by age groups 50-69, 70-79 and 80+ years in women with invasive breast cancer treated by BCS+RT or Mx-RT 2008-2017 in Sweden.

|  | **50-69 years**  **N=21839** | **70-79 years**  **N=8372** | **80+ years**  **N=3928** | **Overall**  **N=34139** | ***P* value** |
| --- | --- | --- | --- | --- | --- |
|  | **N (%)** | **N (%)** | **N (%)** | **N (%)** |  |
| **Follow-up (years), median (range)** | 6.58 [0.09-11.70] | 5.66 [0.10-11.69] | 4.64 [0.09-11.66] | 6.14 [0.09-11.70] | <0.001 |
| **Year of surgery** |  |  |  |  | <0.001 |
| 2008-2009 | 4106 (18.8) | 1285 (15.3) | 747 (19.0) | 6138 (18.0) |  |
| 2010-2011 | 4887 (22.4) | 1670 (19.9) | 858 (21.8) | 7415 (21.7) |  |
| 2012-2013 | 5059 (23.2) | 1934 (23.1) | 973 (24.8) | 7966 (23.3) |  |
| 2014-2015 | 5133 (23.5) | 2237 (26.7) | 901 (22.9) | 8271 (24.2) |  |
| 2016-2017 | 2654 (12.2) | 1246 (14.9) | 449 (11.4) | 4349 (12.7) |  |
| **Tumor stage^** |  |  |  |  | <0.001 |
| T1 | 16889 (77.3) | 5925 (70.8) | 1618 (41.2) | 24432 (71.6) |  |
| T2 | 4773 (21.9) | 2348 (28.0) | 2075 (52.8) | 9196 (26.9) |  |
| T3 | 177 (0.8) | 99 (1.2) | 235 (6.0) | 511 (1.5) |  |
| **Nodal stage^** |  |  |  |  | <0.001 |
| N0 | 16821 (77.0) | 6554 (78.3) | 2245 (57.2) | 25620 (75.0) |  |
| N1 | 3912 (17.9) | 1323 (15.8) | 833 (21.2) | 6068 (17.8) |  |
| N2 | 486 (2.2) | 174 (2.1) | 217 (5.5) | 877 (2.6) |  |
| N3 | 194 (0.9) | 78 (0.9) | 89 (2.3) | 361 (1.1) |  |
| Missing | 426 (2.0) | 243 (2.9) | 544 (13.8) | 1213 (3.6) |  |
| **Region of residence** |  |  |  |  | <0.001 |
| Stockholm/Gotland | 4792 (21.9) | 1797 (21.5) | 704 (17.9) | 7293 (21.4) |  |
| Uppsala/Örebro | 4529 (20.7) | 1687 (20.2) | 904 (23.0) | 7120 (20.9) |  |
| North | 2144 (9.8) | 829 (9.9) | 325 (8.3) | 3298 (9.7) |  |
| South | 4045 (18.5) | 1542 (18.4) | 822 (20.9) | 6409 (18.8) |  |
| Southeast | 2118 (9.7) | 845 (10.1) | 385 (9.8) | 3348 (9.8) |  |
| West | 4211 (19.3) | 1672 (20.0) | 788 (20.1) | 6671 (19.5) |  |
| **Primary treatment** |  |  |  |  | <0.001 |
| Primary surgery | 21505 (98.5) | 8264 (98.7) | 3827 (97.4) | 33596 (98.4) |  |
| Primary systemic treatment | 334 (1.5) | 108 (1.3) | 101 (2.6) | 543 (1.6) |  |
| **Locoregional treatment** |  |  |  |  | <0.001 |
| BCS+RT | 17762 (81.3) | 5404 (64.5) | 649 (16.5) | 23815 (69.8) |  |
| Mastectomy-RT | 4077 (18.7) | 2968 (35.5) | 3279 (83.5) | 10324 (30.2) |  |
| **Axillary surgery*** |  |  |  |  | <0.001 |
| Axillary lymph node dissection | 4583 (21.0) | 1651 (19.7) | 1216 (31.0) | 7450 (21.8) |  |
| Sentinel lymph node biopsy only | 17058 (78.1) | 6575 (78.5) | 2183 (55.6) | 25816 (75.6) |  |
| Missing | 198 (0.9) | 146 (1.7) | 529 (13.5) | 873 (2.6) |  |
| **Histological invasive tumor type** |  |  |  |  | <0.001 |
| Ductal | 17469 (80.0) | 6316 (75.4) | 2876 (73.2) | 26661 (78.1) |  |
| Lobular | 2503 (11.5) | 1249 (14.9) | 576 (14.7) | 4328 (12.7) |  |
| Other | 1467 (6.7) | 665 (7.9) | 369 (9.4) | 2501 (7.3) |  |
| Missing | 400 (1.8) | 142 (1.7) | 107 (2.7) | 649 (1.9) |  |
| **Nottingham histological grade^** |  |  |  |  | <0.001 |
| Grade 1 | 5486 (25.1) | 1823 (21.8) | 608 (15.5) | 7917 (23.2) |  |
| Grade 2 | 10699 (49.0) | 4469 (53.4) | 2020 (51.4) | 17188 (50.3) |  |
| Grade 3 | 5103 (23.4) | 1887 (22.5) | 1151 (29.3) | 8141 (23.8) |  |
| Missing | 551 (2.5) | 193 (2.3) | 149 (3.8) | 893 (2.6) |  |
| **Subtype^** |  |  |  |  | <0.001 |
| HR+HER2- | 15456 (70.8) | 6137 (73.3) | 2547 (64.8) | 24140 (70.7) |  |
| HR+HER2+ | 1503 (6.9) | 449 (5.4) | 206 (5.2) | 2158 (6.3) |  |
| HR-HER2+ | 447 (2.0) | 126 (1.5) | 70 (1.8) | 643 (1.9) |  |
| HR-HER2- | 1055 (4.8) | 393 (4.7) | 225 (5.7) | 1673 (4.9) |  |
| Missing | 3378 (15.5) | 1267 (15.1) | 880 (22.4) | 5525 (16.2) |  |
| **Chemotherapy^+^** |  |  |  |  | <0.001 |
| Yes | 6134 (28.1) | 1210 (14.5) | 51 (1.3) | 7395 (21.7) |  |
| No | 15705 (71.9) | 7162 (85.5) | 3877 (98.7) | 26744 (78.3) |  |
| **Endocrine treatment^+^** |  |  |  |  | <0.001 |
| Yes | 13828 (63.3) | 5582 (66.7) | 2562 (65.2) | 21972 (64.4) |  |
| No | 8011 (36.7) | 2790 (33.3) | 1366 (34.8) | 12167 (35.6) |  |
| **Anti-HER2 therapy^+^** |  |  |  |  | <0.001 |
| Yes | 1761 (8.1) | 389 (4.6) | 37 (0.9) | 2187 (6.4) |  |
| No | 20078 (91.9) | 7983 (95.4) | 3891 (99.1) | 31952 (93.6) |  |
| **Highest level of education** |  |  |  |  | <0.001 |
| ≤ 9 years | 4066 (18.6) | 2857 (34.1) | 2098 (53.4) | 9021 (26.4) |  |
| 10-13 years | 9827 (45.0) | 3256 (38.9) | 1194 (30.4) | 14277 (41.8) |  |
| > 13 years | 7777 (35.6) | 2139 (25.5) | 579 (14.7) | 10495 (30.7) |  |
| Missing | 169 (0.8) | 120 (1.4) | 57 (1.5) | 346 (1.0) |  |
| **Family status** |  |  |  |  | <0.001 |
| Married/partner | 13569 (62.1) | 4505 (53.8) | 1022 (26.0) | 19096 (55.9) |  |
| Single | 8220 (37.6) | 3853 (46.0) | 2897 (73.8) | 14970 (43.9) |  |
| Missing | 50 (0.2) | 14 (0.2) | 9 (0.2) | 73 (0.2) |  |
| **Family income** |  |  |  |  | <0.001 |
| Low | 3688 (16.9) | 3179 (38.0) | 2564 (65.3) | 9431 (27.6) |  |
| Middle | 11842 (54.2) | 4447 (53.1) | 1233 (31.4) | 17522 (51.3) |  |
| High | 6274 (28.7) | 737 (8.8) | 131 (3.3) | 7142 (20.9) |  |
| Missing | 35 (0.2) | 9 (0.1) | 0 (0) | 44 (0.1) |  |
| **Country of birth** |  |  |  |  | <0.001 |
| Sweden | 18866 (86.4) | 7402 (88.4) | 3565 (90.8) | 29833 (87.4) |  |
| Europe, not Sweden | 2183 (10.0) | 847 (10.1) | 320 (8.1) | 3350 (9.8) |  |
| Outside of Europe | 782 (3.6) | 122 (1.5) | 43 (1.1) | 947 (2.8) |  |
| Missing | 8 (0.0) | 1 (0.0) | 0 (0) | 9 (0.0) |  |
| **Charlson Comorbidity Index** |  |  |  |  | <0.001 |
| 0 | 19868 (91.0) | 6882 (82.2) | 2667 (67.9) | 29417 (86.2) |  |
| ≥1 | 1971 (9.0) | 1490 (17.8) | 1261 (32.1) | 4722 (13.8) |  |

BCS; breast-conserving surgery. RT; radiotherapy.

* final axillary intervention i.e. sentinel lymph node biopsy followed by axillary lymph node dissection is classified as axillary lymph node dissection

^from preoperative core needle biopsy in case of neoadjuvant systemic treatment, from surgical specimen in case of primary surgery

**^+^**treatment received in the adjuvant and/or neoadjuvant setting
